# The complete chloroplast genome sequence of a narrow alpine endemic*, Taraxacum hallaisanense* (Asteraceae), on Jeju Island, Korea

**DOI:** 10.1080/23802359.2021.1899072

**Published:** 2021-03-18

**Authors:** Woong Lee, Young-Soo Kim, Seung-Chul Kim, Jae-Hong Pak

**Affiliations:** a Research Institute for Dok-do and Ulleung-do Island, Kyungpook National University, Daegu, Republic of Korea; b Department of Biology, School of Life Sciences, Kyungpook National University, Daegu, Republic of Korea; c Department of Biological Sciences, Sungkyunkwan University, Suwon, Republic of Korea

**Keywords:** Chloroplast genome, alpine endemic, *Taraxacum hallaisanense*, Jeju Island

## Abstract

The first complete chloroplast genome sequence of Jeju Island endemic diploid dandelion, *Taraxacum hallaisanense*, is reported in this study. The plastome size is 151,554 bp in total length, with one large single copy (LSC; 84,066 bp), one small single copy (SSC; 18,524 bp), and two inverted repeat (IR) regions (IR_a_ and IR_b_, each with 24,482 bp). The overall GC content is 37.7% and the genome contained 129 genes, including 84 protein-coding with 2 pseudogenes (*ycf*1 and *acc*D), 37 transfer RNA, and 8 ribosomal RNA genes. Phylogenetic analysis of 19 representative plastomes of the Asteraceae suggests that *Taraxacum* is monophyletic with strong bootstrap support and also that *T. hallaisanense* is closely related to *T. mongolicum*.

The genus *Taraxacum* (Cichorieae, Asteraceae) includes approximately 2500 species, distributing mainly in the northern hemisphere with main diversity in mountains of Eurasia (Richards 1973; Ge et al. 2007). A few species also occur in the temperate regions of the southern hemisphere (Ge et al. 2007). *Taraxacum* represents an example of evolutionary and taxonomic complexity due to agamospermous reproduction, complex multiple hybridity, and frequent polyploidy (Asker and Jerling 1992; Kirschner et al. 2003, 2015; Záveská Drábková et al. 2009). In East Asia, approximately 50 species in two sections (*Mongolica* and *Ceratophora*) can be found with one center of diversity in Japan (22 species) (Kitamura 1957; Morita 1980). Six species of *Taraxacum* occur in the Korean Peninsula: native *T. coreanum* and introduced *T. officinale* distribute widely throughout all provinces, while two natives *T. ohwianum* and *T. platypecidum* rather narrowly occur in central and northeastern parts of the Korean Peninsula. *Taraxacum hallaisanense* is very narrowly restricted to Jeju Island, whereas *T. mongolicum* occurs disjunctly in North and South Korea. While a baseline phylogenetic framework of *Taraxacum* was hypothesized based on the internal transcribed spacer of nuclear ribosomal DNA (nrDNA ITS) and chloroplast DNA sequences (Kirschner et al. 2003, 2015), we know very little about overall phylogenetic relationships among species in East Asia. With regard to complete plastid genome sequences of *Taraxaxum* in Korea, two traditional medicinal herbs, *T. platycarpum* and *T. mongolicum*, were reported (Kim et al. 2016). However, little is known for complete plastid genome sequences of congeneric species in Korea, such as *T. coreanum*, *T. ohwianum*, *T. platypecidum*, etc. Therefore, the aim of this study is to characterize the plastid genome of *T. hallaisanense*, which is endemic to Jeju Island, Korea, and determine its phylogenetic position among congeneric species.

Total DNA (Voucher specimen: 33°21′31′′N, 126°30′11′′E; 1600 m elevation) was isolated using the DNeasy plant Mini Kit (Qiagen, Carlsbad, CA) and sequenced by the Illumina platform (Macrogen, Seoul, Korea). The specimen was deposited at Kyungpook National University Herbarium (KNU; http://bio.knu.ac.kr/PhD/profile/lst.do?seq=6, Jae-Hong Pak, jhpak@knu.ac.kr) under the voucher specimen of 'Lee180531-001'. A total of 30,937,892 paired-end reads were obtained and assembled *de novo* with Velvet v. 1.2.10 using multiple *k*-mers (Zerbino and Birney 2008). The tRNAs were confirmed using tRNAsacn-SE (Lowe and Eddy 1997). The complete plastome length of *T. hallaisanense* (MW067130) was 151,554 bp, with one large single-copy region (LSC; 84,066 bp), one small single-copy region (SSC; 18,524 bp), and two inverted repeat regions (IR_a_ and IR_b_; 24,482 bp each). The overall GC content was 37.7% and the plastome contained 129 genes, including 84 protein-coding genes with 2 pseudogenes (*ycf*1 and *acc*D), 8 rRNA, and 37 tRNA genes. A total of 14 genes were duplicated in the inverted repeat regions, including 7 tRNA, 4 rRNA, and 3 protein-coding genes.

Nineteen representative species of Asteraceae (18 from tribe Cichorieae and one outgroup from tribe Astereae), including *T. hallaisanense*, were aligned using MAFFT v.7 (Katoh and Standley 2013) and based on complete plastid genome sequences, maximum likelihood (ML) analysis with 1000 bootstrap replications was conducted using IQ-TREE v.1.6.7 (Nguyen et al. 2015). *Aster tataricus* (tribe Astereae) was used as an outgroup. The ML tree suggested that the genus *Taraxacum* is monophyletic (100% bootstrap support, BS) (Figure 1). *Taraxacum* is sister to the clade containing closely related genera, such as *Crepidiastrum*, *Lapsanastrum*, and *Youngia* (100% BS). Within the genus *Taraxacum*, two major lineages can be identified; one lineage includes *T. hallaisanense* and *T. mongolicum* and the other includes *T. amplum*, *T. officinale*, *T. obtusifrons*, and *T. platycarpum* (Figure 1). The plastid genome suggested that *T. hallaisanense*, which is a narrow Jeju Island endemic and diploid (2*n* = 2*X* = 16) species in Korea, is closely related to *T. mongolicum*, which is triploid (2*n* = 3*X* = 24) occurring in the mainland. However, species relationships among diploid species in Korea and Japan based on 15 capitulum traits suggested that Korean species, *T. hallaisanense* and *T. ohiwanum* (diploid and triploid) are related to *T. japonicum*, which occurs in southern-central part of Honshu, Japan (Lee et al. 2004). To infer accurate phylogenetic relationships among species of diploid and polyploid *Taraxacum* species in East Asia, it is necessary to sample broadly and apply highly variable nuclear and chloroplast markers. The complete plastid genome assembled in this study will provide valuable information for identifying useful plastid markers for DNA barcoding and elucidating phylogenetic among species within the genus.

**Figure 1.**
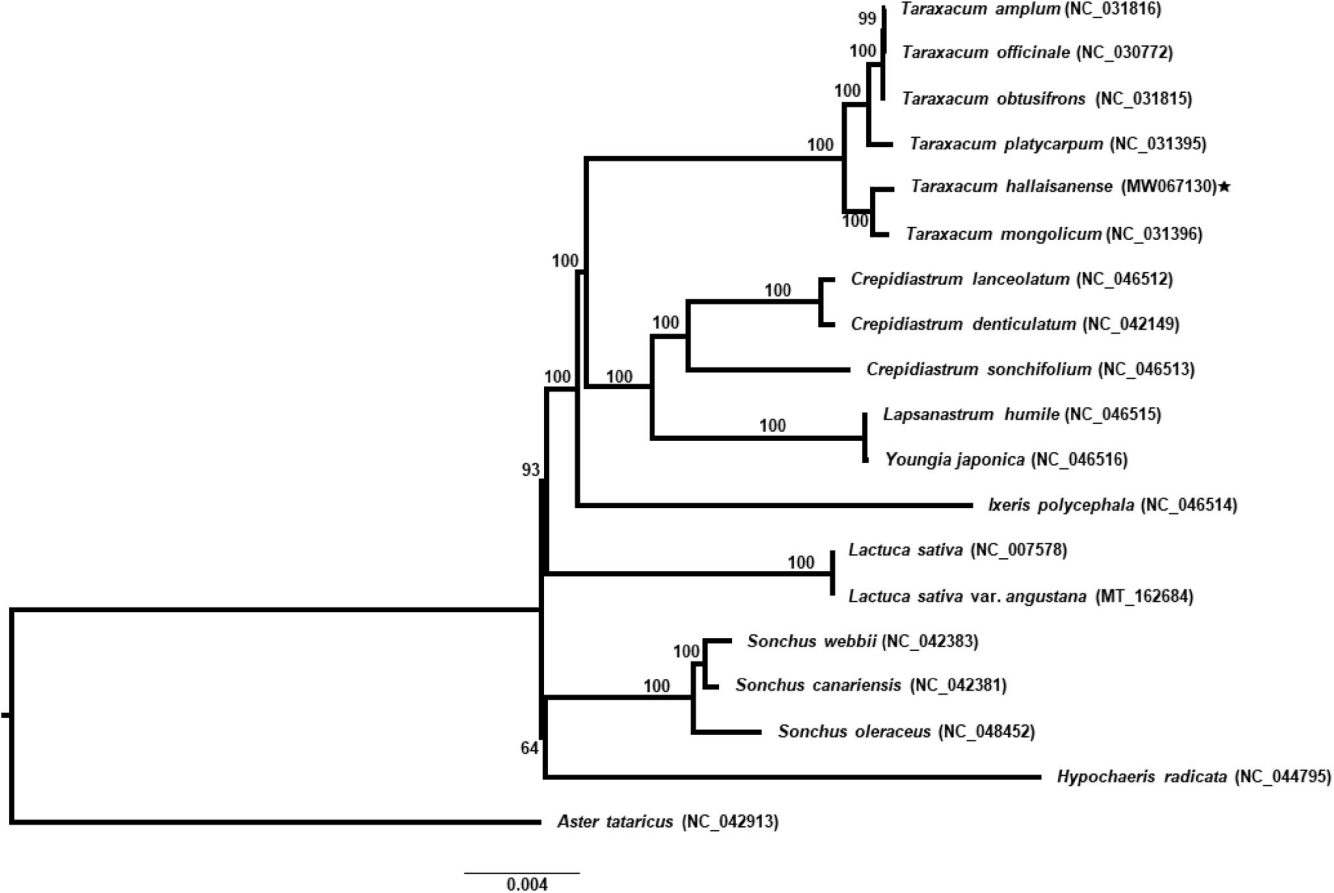
The maximum-likelihood (ML) tree based on 18 representatives of Asteraceae (tribe Cichorieae) and one outgroup taxon, *Aster tataricus* (tribe Astereae). The bootstrap support value based on 1000 replicates is shown on each node.

## Data Availability

The genome sequence data that support the findings of this study are openly available in GenBank of NCBI at https://www.ncbi.nlm.nih.gov/ under the accession no. MW067130. The associated BioProject, SRA, and Bio-Sample numbers are PRJNA697908, SRR13590697, and SAMN17676085, respectively.
